# Supplementary material for: Chromothripsis during telomere crisis is independent of NHEJ, and consistent with a replicative origin
Source: Genome Res. 2019 May;29(5):737–49. doi: 10.1101/gr.240705.118 (PMC6499312; doi:10.1101/gr.240705.118)
Supplement: Supplemental Material [file supp_gr.240705.118_Supplemental_file_1.zip › contigs/annotated_contigs/DB113/contig.2.DB113_length_625_mean_cov_16.08.docx]

**DB113_length_625_mean_cov_16.08**

```
ACCATTGTACTCCAGCCTGGCCAACAAGAGCAAAACTCCGTCTCAAAAAAAAAAAGAAAAGAAAAGAAAACTGATTTCTCAGTTCAGGC
 >chr19:32461700-32462004 + E=5e-167
TATCTCTTCTCTCTGTTTGTAACACAAAAGACTTATGCCTCTGAAGGACGTTGTAGAGAGAATATTGCGATGGGAAACATACAACTTTG

AATTTCATTACATCACAAACTTCCTTCTCAAAAATATAACACCAGTTAGTTTAATAAAACATGGCATCTACGGACATACTTATCATGCA

GAGGTGCAATAAGAAACAATCATATTAACTGAAAACCA|AGCTCTTCCTTTCCCAGAGGGCTGGAGCCGATGGCCTCGATGACAGCCTA
                                        >chr19:32463793-32464113 + E=8e-181
GGCATTCAGTTGGCTTCGCCTGGAGGAAGAGGGAGGCAAACGTGCCTGAATGATTCTCCCCAGGAAAGGGAGAGAGTAGGACAACTTGG

GGAAAAAACGTTCCTGCAGGGCTGCAAAAATCAGGGAGAGGCGGCAGGGCAAGGAGACAGGCGTATGTATGTCTGTGTCTTTCAGCTAG

GCAGCTCAATCTCAGGTTATTTTATTTGACAAATAAAACTAAACTGCATATTTATGGACTAATGATGTGAGATATTGCTCTTTTATTCG

TAT
```
